# A Department of Public Health1Speech delivered in the Senate, March 24, 1910.

**Published:** 1910-05

**Authors:** R. L. Owen

**Affiliations:** United States Senator


### BUFFALO MEDICAL JOURNAL.

VOL. LXV. MAY, 1910. NO. 10

### ORIGINAL COMMUNICATIONS.

### A Department of Public Health[1]

BY HON. R. L. OWEN, United States Senator.

MR. PRESIDENT, the people of the United States suffer a loss of over 600 000 lives per annum. This terrible loss might be prevented by reasonable safeguards under the coöperation of the federal and state authorities, each within strict constitutional limits.

These deaths are caused by polluted water, impure and adulterated food and drugs, epidemics, various preventable diseases—tuberculosis, typhoid and malarial fevers, and so forth—unclean cities, and bad sanitation.

Measuring the money value of an American citizen at $1,700, this preventable loss by death is one thousand millions of dollars annually, equal to the gross income of the United States Government.

There are 3,000,000 people in the United States on the sick list from preventable causes, of whom 1,000,000 are in the working period of life; about three-quarters of a million actual workers losing on an average of $700 per annum, an approximate loss from illness of five hundred millions, and adding a reasonable allowance for medicine, medical attendance, special food and care, a like sum of five hundred millions, these losses would make another thousand million dollars of preventable loss to the people of the United States.

AUTHORITY FOR FACTS STATED.

Do you imagine that these figures are exaggerated or fanciful, Mr. President? They are confirmed to us by the report of the Committee of One Hundred on National Health in its Report on National Vitality. (Bulletin No. 30, p. 12.) This bulletin was prepared by Professor Irving Fisher, professor of political economy of Yale University, with the assistance of the most learned men in the whole world, including Professor Lafayette

1. Speech delivered in the Senate, March 24, 1910.

B. Mendel, of Sheffield Scientific School of Yale University; Professor M. V. O'Shea, University of Wisconsin; Dr. Charles W. Stiles, a chief of the hygienic laboratory of the United States Public Health and Marine-Hospital Service; Robert M. O'Reilly, former Surgeon-General of the United States Army; Professor C. I. Henderson, University of Chicago; and the officials of the various public health societies and of the American Medical Association; Dr. George M. Kober, dean of the Georgetown Medical College; Dr. Norman E. Ditman, Columbia University; Hiram J. Messenger, actuary of the Travelers' Insurance Company, and so forth.

Our pension roll of over $150,000,000 per annum is three-fourths of it due to illness and death from diseases that were preventable. Under a wise administration in the past the United States would today be saving an annual charge of about $125,000,000 on the pension list, and would have saved under this heading over $2,000,000,000 and much human misery and pain.

Will you fail to listen when on my honor as a Senator I call your attention to the vast importance of this matter and the high standing of those who vouch for the accuracy and reliability of this statement? Will you, as the representatives of the people of the United States, fail to investigate and to act in a matter of such consequence?

### ORIGIN OF BILL 6049.

There are the facts; there are the authorities. Mr. President, nine years ago I had the importance of this subject called to my attention by an article read before the Cincinnati Academy of Medicine, October 7, 1901, on "Preventable disease in the Army of the United States—cause, effect, and remedy," by Major William O. Owen, a surgeon in the United States Army, printed in the *Journal of the American Medical Association,* October 26, 1901, where he pointed out over 19,000 cases of typhoid fever in four camps—Chickamauga, Alger, Meade, and Jacksonville—with 1,460 deaths of the finest young men of America, nearly all of which was a preventable loss. The typhoid cases, with resultant deaths, were due to ignoring the laws of sanitation. (Exhibit 10.) I drew this bill (S. 6049) in the hope of coöperating with the administration in making effective the most important of all forms of conservation—the conservation of human life—and in the hope of making effective the expressed desires of the numerous associations and societies of the United States who stand for a department of public health.

No man can read the Report on National Vitality—Its Wastes and Conservation, of the Committee of One Hundred without being impressed with certain great facts:

1. The thoroughness and scientific care with which it made this report.

2. The stupendous annual loss of life which could be prevented; the immense economic commercial loss and human misery and sorrow due to preventable illness, inefficiency, degeneration, and death.

3. The wisdom of the means proposed by the Committee of One Hundred for the prevention of this annual loss and for the conservation of the national life and health.

SUPPORT OF THE PLAN PROPOSED.

There is not in the world a more distinguished body of scientists and philanthropists than the Committee of One Hundred, appointed by the American Association for the Advancement of Science.

Irving Fisher, professor of political economy of Yale University is its president. The vice-presidents are: Rev. Lyman Abbott, editor Outlook, New York City; Miss Jane Addams, of Hull House, Chicago; Felix Adler, lecturer, of New York City; James Burrill Angell, diplomat, New York City; Honorable Joseph H. Choate, ex-ambassador to England, New York City; Charles William Eliot, president of Harvard University, Cambridge, Mass.; Right Reverend Archbishop Ireland, St. Paul, Minn.; Honorable Ben B. Linsay, Denver, Colo.; John Mitchell, New York City; Dr. William H. Welch, professor pathology, Johns Hopkins University, Baltimore, Md.; Secretary, Edward T. Devine of the Survey; and the list of 100 contains other names as notable, including Miss Mabel T. Boardman, president of the Red Cross; Andrew Carnegie, Thomas A. Edison, Mrs. John B. Henderson, of Washington; Professor David Starr Jordan, president Stanford University; Dr. Charles A. L. Reed, chairman of the legislative committee of the American Medical Association, of Cincinnati, Ohio; Robert S. Woodward, president Carnegie Institution, Washington, D. C.; and a host of others no less distinguished for learning, patriotism, and philanthropy.

INCREASING LENGTH OF LIFE.

The modern duration of life is widely variant, according to the organised protection of the health of the people by government.

In India the average length of life is twenty-three years, due, not to climatic conditions, but to ignorance, prejudices, and religious superstitions. They will not kill a snake in India, and thousands of inhabitants die annually from the poison of snake bites. In America we die in like manner from typhoid and tuberculosis, because we neglect to suppress the causes of these diseases.

The length of life in India is not increasing because of their lack of progress; but in Geneva, Switzerland, where the country is supposed to be very healthy, the length of life in the sixteenth century was only 21.2; in the seventeenth century, 25.7; in the eighteenth century, 33.6; from 1801 to 1883, 39.7; and it is steadily improving.

### THE PROLONGATION OF LIFE.

Scientific hygiene and increased knowledge of the laws relating to health have had a very striking effect upon the prolongation of human life throughout the world.

At present in Massachusetts life is lengthening at the rate of fourteen years per century; in Europe about seventeen years; in Prussia, the land of medical discovery and its application, twenty-seven years; in India, where medical progress is practically unknown, the life span is short, twenty-three, and remains stationary.

It is demonstrated beyond reasonable doubt by the report of the Committee of One Hundred that the average human life in the United States may be, within a generation, prolonged over fourteen years. I submit the table as to the method of this calculation.[1]

This detailed estimate of the prolongation of human life, fourteen years, is based upon a vast amount of data and is a conclusion justified by the knowledge of the most learned men in the world.

I remind you again of what I pointed out a year ago, that in New Zealand the deaths per thousand per annum is 9 and a fraction and in the Australian states 10 and a fraction, while in the United States it is 16.5, a loss of 7 to the thousand in the United States in excess of the New Zealand rate—that is, in 90,000,000 people it would exceed 600,000 deaths that could be saved annually in our Republic.

### YELLOW FEVER.

Before the American intervention in Cuba the death rate from yellow fever alone in Habana to the hundred thousand population in 1870 was 300; in 1880, 324; in 1896, 639; in 1897, 428; and after the American occupation it fell: 1900, 124; in 1901, 6; in 1902, zero; in 1903, zero; in 1904, zero.

1. This table is omitted for want of space.

What a glorious record! What a splendid tribute to the learning, industry, and self-sacrifice of the devoted medical men who accomplished this result, most of whom are now dead. James Carrol and Lazier died from experimental yellow fever, sacrificing their own lives deliberately in the interest of their fellow-man. All honor to their names and to the names of Walter Reed and the others, who, brave, gallant soldiers of peace, exposed their lives for the benefit of their fellows. Monuments of stone and of bronze should be erected to these noble patriots of peace, more noble and self-sacrificing in their work than patriots of war. What does the commerce of the world owe to these men who vanquished yellow fever? There could have been no Panama Canal except for this development of science.

### HOOKWORM.

I am informed by a high authority that over 90 per cent. of the children of one of the Southern States are afflicted with the hookworm, a preventable disease, curable at a cost of less than 50 cents apiece with two doses of thymol and a little careful treatment, yet the disease is denied, prejudices and lack of learning stand in the way of the speedy restoration of thousands, and the voice of the men who know the habits, life, history, and remedy for hookworm carries with it little power or authority to heal the unlearned patients. Only the power of the State itself, with its dignity and with its authority; only the power of the General Government, with its prestige and with its high standing, is competent to impress upon the minds of the unlearned body of the people those principles of hygiene, preventive medicine, and sanitary law which are essential to the preservation of the life of the American people.

### PEOPLE UNINFORMED EXPOSE THEMSELVES.

With the record in Habana of the control of yellow fever there are thousands of unlearned people who will ignorantly ridicule the means of the mosquito as an agency for transmitting this disease; that will deny the transmission of malaria by the mosquito.

And there are thousands who will ignorantly deny that bubonic plague is transmitted by the flea from the rat and the squirrel to the human being. The power of the Government alone acting through its strongest arm is necessary for the prevention of a wholesale introduction into the United States of bubonic plague.

The bubonic plague is now among the ground squirrels and rats on the Pacific coast at various scattered points over a thousand miles apart, due to the thoughtless ignorance, interest, and prejudice of the commercial interests of San Francisco that suppressed the faithful and intelligent work attempted to be discharged by the officers of the Marine-Hospital Service, which I may more fully set up hereafter.

The bill which I have introduced is in accordance with the earnest repeated desires of the American Medical Association, probably the largest and most honorable association of physicians and surgeons in the whole world. As far as the principle of the bill is concerned, I have an earnest letter from Dr. Charles A. L. Reed, chairman of the legislative committee of the American Medical Association, which I herewith insert:

CINCINNATI, March 10, 1910.

HON. ROBERT L. OWEN,

United States Senate, Washington, D. C.

DEAR SIR—In compliance with your request for suggestion to be taken up in connection with the hearing on the bill recently introduced by you to create a department with a secretary of health, I beg to reply in my capacity as chairman of the legislative committee of the American Medical Association. In that capacity I have the honor at the same time to request, first, that you avail yourself of an early opportunity, and in your own way, to lay before the Senate the facts which I shall present; and, second, that you arrange at an early date for a hearing on your bill, the vital principle of which is so distinctly in consonance with the interests of the people, as represented by and through the medical profession.

This is shown by the fact that the American Medical Association, through its legislative conference, attended by delegates from 36 States and from the army, navy, and the Public Health and Marine-Hospital Service, held at Chicago, March 2, 1910, urged by resolution, as the association has repeatedly urged for nineteen years, "that a bill be passed recognising the health interests of the country in the title of a department of the National Government, and that within that department there be organised all national health agencies."

The physicians of the country, who, as professional students of the question and as the natural advisors of the people on health questions, and who, consequently, have first knowledge of the subject, have long maintained their present attitude for the following specific reasons:

First—The time has arrived when, under the law of precedent, the health interests of the country ought to pass from their present bureau stage of development to that of a department. This course of evolution was exemplified, first, I believe, in the development of the Department of the Interior, then that of Agriculture, and, finally, that of Commerce and Labor. In each of these instances the antecedent bureaus had existed for periods varying from a few years to a decade or two. The health interests of the country, more fundamental than all, have been left in the form of, successively, a "service," then of a "bureau," for more than a century.

Second—The creation of a department of health is furthermore demanded: first, because sanitary science has demonstrated its ability to conserve the efficiency and prolong the life of the people; and, second, because nothing less than the establishment of a department can have that maximum of moral force and educational influence, that maximum of prestige and effectiveness combined with business-like economy of administration that will enable it to deal with the disgraceful, not to say monstrous, conditions now prevailing in this country.

Third—That a department of health, with the fullness of power and influence that can inhere only in a department and nothing less than a department, is demanded by the conditions to which I have alluded is conclusively established by the fact that, first, about 600,000 people die in this country every year from preventable causes; second, that something more than $3,000,000 more are made ill and idle for variable periods every year from the same causes; and, third, that the annual economic loss from this source alone amounts to more than a billion and a half dollars every year.

Fourth—That nothing less than a department of health, acting in coöperation with the States and in full recognition of their rights and powers, is practicable for the assembling and coordinating of the existing health agencies of the Government and for their effective, economic, and business-like administration.

Fifth—That nothing less than the creation of a department of health can comprise a fulfillment of the pledge to the people contained in the platform of every political party that appealed to the popular suffrage in the last national campaign.

In view of the foregoing facts and considerations I have the honor to request that at the hearing on your bill, care be taken to give special consideration to the suggestions which I shall enumerate.

Many, if not all of them, have been covered in general terms and some of them in specific terms, in your bill. It has seemed, however, that by presenting them somewhat in detail in the form of sections to a possible bill, I could facilitate their consideration in consecutive order as follows:

Section 1 ought to provide, as your bill does provide, for the establishment of a department of health under the supervision of the secretary of health, who shall be appointed by the President by and with the consent of the Senate, at a salary of $12,000 per annum and who shall be a member of the Cabinet of the President and who shall discharge the duties prescribed in the act.

Section 2 might with propriety provide for the constituent bureaus of the Department of Health as follows:

(*a*) The Bureau of Hygiene and Preventive Medicine, to which (a) shall be transferred the Laboratory of Hygiene, now located in the Bureau of Public Health and Marine-Hospital Service in the Department of the Treasury, together with all duties, functions, powers, rights, and prerogatives now vested by law in such Laboratory of Hygiene; and it shall be the further duty of the Bureau of Hygiene and Preventive Medicine (b) to coöperate with the respective States, Territories, and dependencies in accumulating statistics and other information as to causes and prevalence of disease; (c) to conduct continuous investigation into all sources of danger to human health and life; (d) to formulate rules and regulations for carrying out these provisions, and (e) to publish the records and results of its labors, all under the direction and by the approval of the Secretary of Health.

(*b*) The Bureau of Foods and Drugs, to which (a) shall be transferred all duties, functions, powers, rights, and prerogatives now devolving by the Food and Drugs Act of 1907 on the Bureau of Chemistry of the Department of Agriculture; and the Bureau of Foods and Drugs shall also (b) supervise the cleanliness and other hygienic and sanitary features of the buildings and products of manufactories, cold-storage plants, and other establishments engaged in the commercial preparation or in the storage of any food product or products whatsoever destined for interstate commerce; (c) establish standards of purity of foods; (d) conduct investigations to determine the best method of preparing foods with reference to the full development of their nutritive value; (e) determine the food value of articles not now generally recognised as foods; (f) establish standards of purity for drugs; (g) make a systematic and exhaustive study of the medicinal flora of the United States and its Territories and dependencies; (h) investigate and, where practicable, promote the naturalisation and commercial cultivation within the United States, its Territories and dependencies, of medicinal flora indigenous to other countries; (i) publish reports of its investigations, activities, and conclusions; and (j) formulate and enforce necessary rules and regulations all under the direction of the Secretary of Health.

(*c*) The bureau of marine hospitals, to which shall be transferred the Marine-Hospital Service of the Bureau of Public Health and Marine-Hospital Service of the Department of the Treasury, together with its present personnel and all duties, functions, powers, rights, and prerogatives now vested by law in such Marine-Hospital Service, all to be administered under the direction of the secretary of health.

(*d*) The bureau of quarantine, to which shall be transferred the Quarantine Service now located in the Bureau of Public

Health and Marine-Hospital Service of the Department of the Treasury, together with its present personnel and all duties, functions, powers, rights, and prerogatives now vested by law under such Quarantine Service, all to be administered under the direction of the secretary of health.

(*e*) The bureau of institutions and reservations, to which shall be transferred all hospitals, asylums, "homes," and infirmaries located in any other department of the Government except the Department of War and the Department of the Navy. And there shall likewise be transferred to this bureau the Hot Springs Reservation and all other reservations now or hereafter established by the Federal Government for the conservation of health.

(*f*) The bureau of vital statistics, to which shall be transferred the Bureau of Vital Statistics now located in the Department of Commerce and Labor, together with its present personnel and all duties, functions, powers, rights, and prerogatives now vested by law in such Bureau of Vital Statistics.

(*g*) The bureau of publication and publicity, which shall (a) publish the reports of the secretary of health and all reports, bulletins, and documents of all bureaus of the department of health when approved for the purpose by the secretary of health, and (b) devise and carry out the most effective means by which information originating in the department of health or any of its bureaus may be most widely and effectively disseminated for the information and guidance of the people.

Section 3 might with equal propriety provide that (a) there shall be a medical service of the Department of Health (b) designated by the initials U. S. H. S., meaning "United States Health Service." (c) which service shall consist of (1) a Regular Medical Corps, which shall consist of the United States Marine-Hospital Corps with its present personnel and without other modification in the law governing the same, or in the regulations enacted in pursuance of such law than may be necessary to comply with the provisions of this act. (2) A special Medical Corps, which shall consist of all physicians, surgeons, and medical officers now employed in any capacity in any department of the Government, excepting in the army and navy who, subject to the direction of the secretary of health, but without having their status otherwise disturbed, shall continue in their present capacity until the expiration of their present tenure, but thereafter all such positions shall be filled by detail from the regular Medical Corps which shall be selected in the first instance in accordance with regulations not less exacting than those which now govern entrance into the Marine-Hospital Corps. (d) The secretary of health shall, consistently with the provisions of this act, (1) define the grades of health service with due regard to the period of service and efficiency record of its members: (2) prescribe uniforms and insignia for each grade; (3) formulate rules and regulations for the government of the corps, and at his discretion (4) detail any member of the corps for duty in any bureau of the Department of Health, or (5) for duty in any other department on request of the secretary of such department, or (6) for duty in any State, Territory, or dependency, or in the Panama Canal Zone when requested so to do by the proper authority of such State, Territory, dependency, or the Panama Canal Zone whenever the resources of the service will permit such detail.

Section 4 might further define the duty of the secretary of health by stating that in addition to the duties elsewhere prescribed in the act (a) he may, in his discretion, transfer specific duties from one bureau to the other whenever required in the interests of both economy and efficiency; (b) exercise all the functions heretofore exercised, respectively, by the Secretary of the Treasury, the Secretary of the Interior, the Secretary of Agriculture, and the Secretary of Commerce and Labor in connection with any bureau, division, or service transferred by the act to the Department of Health; (c) exercise all duties heretofore exercised by the Secretary of Agriculture in the enforcement of the pure food and drugs act; (d) discharge such other duties as may be prescribed from time to time by the President and, finally, (e) prepare and submit reports relative to his department embracing suggestions for the improvement of its service, including recommendations for change in personnel, duties, and salaries.

Section 5 might provide (a) that the President be authorised and directed within one year from the passage of the act to appoint an advisory board of health to consist of six members, two to be appointed for one year, two for two years, and two for three years each, who shall serve without pay, except their traveling expenses, for not more than six meetings annually, and whose functions shall be to confer with and advise the secretary of health relative to all questions of policy pertaining to human health and upon other questions at the request of the secretary of health; (b) the present consultative arrangement between the present Bureau of Health and representatives of the state boards of health might with propriety be continued between the Department of Health, its Secretary, advisory health boards, chiefs of bureaus, and the representatives of the state boards of health.

Section 6 and succeeding sections might provide in the usual way for the transfer of officers, clerks, employees, property, fixtures, etc.

In asking that you take the foregoing points under special consideration; that the hearing be arranged for the earliest practicable date, and that legislation be reached, if possible, at the present session of Congress, may I ask that you urge upon your colleagues the importance to the people of giving due weight to the conditions to which I have referred?

I have said that over 600,000 of our people die every year from preventable causes. Suppose that our entire army and navy were swept off the earth not once but three times in a year.

Would the Congress do anything about it? There are nearly 5,000,000 needlessly ill every year. Suppose that every man, woman, and child in all New York, with Boston and Washington added, were similarly stricken. Would the Congress inaugurate an inquiry? Our losses from these causes amount to a billion and half dollars every year. Suppose that every dollar appropriated annually for the expense of the Government and half as much more were actually burned up and the ashes blown into the sea. Would the Congress take action in the premises?

Our health agencies are scattered, uncorrelated, and unorganised. Suppose that our monetary system were looked after by a dozen or more bureaus in almost as many departments, and that it were responsible for a billion and a half dollars loss every year. Would the Congress be disposed to think that there was possible relationship between the lack of organisation and the deficit?

In reiterating the request for an early and full hearing on this question, I beg to emphasise the fact that I do so in behalf of the American Medical Association and in behalf of the interests of the people of the United States, as represented by and through the medical profession. And in this behalf and in view of the fact, deducible from our vital statistics, that in this country alone the people are dying from preventable causes at the rate of more than one every minute and that they are falling ill from the same causes at the rate of more than five every minute, may I not venture to suggest that the subject is one of sufficient importance to be entitled to precedence over some other questions that may possibly be engaging the attention of the committee?

Awaiting your early reply, I have the honor to be,

Very sincerely,

CHARLES A. L. REED,

*Chairman of the Legislative Committee,*

*American Medical Association.*

P. S.—I beg leave to advise you that I am sending a letter to the same purport, and largely in the same language as this, to Honorable James R. Mann, of the House, who has requested suggestions to be considered in committee in connection with the recommendations relative to the public-health clause contained in the President's message.

Mr. President, this bill (S. 6049) coördinates and brings into one working body the various health agencies of the Government.

It proposes no new officers except the secretary and his assistant, who should be a permanent officer, acting as a director general. Perhaps such assistant should have this title.

It calls for no new appropriations except the salary of the secretaries.

It will provide a number of economies by preventing duplication, and make more efficient the money expended and the officials employed by the present health agencies of the Government.

The coördination of these agencies has been approved by President Taft, and the vigorous coöperation of such agencies with the state authorities in stamping out disease has been urged by President Roosevelt.

I quote President Taft and what he said in regard to the work of the Committee of One Hundred in their desire to promote the national health:

How nearly this movement will come in accomplishing the complete purpose of its promoters, only the national legislator can tell. Certainly the economy of the union of all health agencies in the National Government in one bureau or department is wise.

President Roosevelt said:

I also hope that there will be legislation increasing the power of the National Government to deal with certain matters concerning the health of our people everywhere. The federal authorities, for instance, should join with all the state authorities in warring against the dreadful scourge of tuberculosis. I hope to see the National Government stand abreast of the foremost state governments.

I introduced this bill providing for a department and not for a bureau. The reason for a department instead of a bureau is perfectly obvious and perfectly unanswerable.

I reiterate and indorse the five substantial reasons given by Dr. Charles A. L. Reed, chairman of the legislative committee of the American Medical Association, and invite special attention to the cogency of the reasons given.

It is generally agreed that these bureaus should all be brought together as one working body. To bring established bureaus under a new "bureau of public health" would be to lower the dignity of the present bureaus by making them the subordinate bureaus of a new bureau, which would be offensive to every bureau so subordinated.

To bring these bureaus under a department would not lower the prestige of a bureau thus coördinated with other bureaus under the department, and would, I believe, generally meet the approval of the government officers employed in the various bureaus so coördinated, giving them a new dignity by being a distinct branch of a department of public health, through which they could enlarge their efficiency and find better expression and publicity of work done for the public health.

We have had bureaus for one hundred years. They are scattered in eight departments. They have been disconnected and without coördination. They have ever been jealous of each other, the one nullifying and hampering the work of another. They have been without a responsible head because of this subdivision and because the chief of the most important of these bureaus, the Surgeon-General of the Public Health and Marine-Hospital Service, can not express an opinion or give information until he has consulted the Secretary of the Treasury.

The Secretary of the Treasury was not selected as a Cabinet officer because of his knowledge of the public health, but because he was an expert on finance. At present our Cabinet expert on finance directs government activities in controlling bubonic plague, and the board of trade and the commercialised physicians of San Francisco would be more important in his eyes in all human probability than the chief of one of his subordinate bureaus; at all events this was true as to a previous Secretary.

### BUBONIC PLAGUE ON THE PACIFIC COAST.

When the bubonic plague broke out in San Francisco in 1900—one of our importations from the Orient, known in former times as the black death or the plague—the city board of health of San Francisco quarantined the Chinese district. (It is the most deadly epidemic disease known to men. In the outbreak in Bombay 220,000 cases gave 164,000 deaths. This disease, as the black death, killed 68,596 people in London in 1665, nearly half of those who remained in the city.) The United States circuit judge, on June 15, 1900, influenced by the commercial spirit of San Francisco, declared the city quarantine illegal, gratuitously observing in his opinion:

If it were within the province of this court to decide the point, *I should hold that there is not and never has been a case of plague in this city.*

This high authority on bubonic plague would also have decided, "if within the province of his court, that there never would be a case in San Francisco." His judgment in the one case would be as persuasive as in the other.

Bubonic plague was then in the city. It is now scattered over the Pacific coast at points a thousand miles apart and is requiring enormous sums of money to stamp it out, and it has not been stamped out, but is now endemic and spreading through the infection of ground squirrels and rats, which continually infect each other and spread the germs of the disease over enlarging areas.

This opinion of the United States circuit judge was followed with an immediate federal quarantine of the State of California, which was the duty of the government officers in charge under the obligation of the United States to the several States of the Union and to the nations of the world. The Marine-Hospital Service officials in San Francisco declared this quarantine.

The governor of California, the Senators of California, and the commercial bodies of San Francisco immediately suppressed the Marine-Hospital Service, compelled the Surgeon-General to yield, proved a false case, and made it temporarily stand as the truth before the country. They furnished evidence and proved (?) that there was no bubonic plague in San Francisco, notwithstanding the fact bubonic plague was there in sober truth.

The Marine-Hospital Service finally persuaded the Secretary of the Interior to cause an inquiry in January, 1901, through experts of the highest class. This unanswerable authoritative report was made on February 26, 1901, finding numerous cases of bubonic plague in the heart of San Francisco. The United States quarantine law (sec. 4) required its immediate publication. It was suppressed until April 19, 1901, and until it had been given publicity by the *Occidental Medical Times*, the *Journal of the American Medical Association*, the *Medical News*, and the *Sacramento Bee*.

Again the commercial interests of San Francisco triumphed over the bureau and compelled the Surgeon-General, the head of the bureau, by an order of his superior officer, the Secretary of the Treasury, to agree to suppress this report, contrary to the obvious moral and sanitary duty of the United States. From that time bubonic plague has widened the area of its terribly dangerous infection from Los Angeles to Seattle, passing from rat to rat and squirrel to squirrel and from these animals to an occasional human being through the agency of the common flea. Various experts of the Marine-Hospital Service, who immediately after the report of 1901 discovered the infection outside of San Francisco and reported the truth, were by some strange fatality shortly after their several reports removed from such duty faithfully peformed and sent to the ends of the world—to Honolulu, to Ecuador, and so forth.

It is a most interesting history, the details of which might with propriety be given to the Senate as showing the destructive power commercial interests can exist over the faithful servants of a subordinate bureau.

The point I wish to emphasise is that the bureau dealing with public health was easily suppressed by commercialism and its supposed interests, putting in jeopardy the national health, the national honor, and the national wealth and treasury and required to withhold and suppress the truth in violation of section 4 of the quarantine laws of the United States.

In 1908 we expended for the suppression of plague, $228,-337.22; in 1909 we expended for the suppression of plague, $337,403,13; for 1910 we appropriated $750,000 and $187,771 unexpended balance—in all, $937,771—for the prevention of epidemics of cholera, typhus and yellow fever, smallpox, and bubonic plague (called also Chinese plague or black death). All of this appropriation was really needed for bubonic plague, which was the only epidemic seriously threatening the United States. Fortunately, we have $724,000 of this on hand. So, from no danger, Mr. President, in 1901, 1902, and 1903, the danger grew to the request for an appropriation of over $900,000 in 1910. There has been over a million dollars appropriated and the plague has not been suppressed. The bureau was prevented giving publicity to the truth, and Mazatlan, Mexico, was infected in consequence of no sufficient precaution.

### OUR INTERNATIONAL OBLIGATIONS.

A department of public health is absolutely essential in order to deal with this matter and with similar questions with the full power and dignity of this Government and in order to faithfully and honorably comply with the state and international sanitary obligations of the United States.

The first article of the first title of the International Sanitary Convention of Paris, 1903, with Germany, Austria-Hungary, Belgium, Brazil, France, Spain, Great Britain, Greece, Italy, Luxemburg, Montenegro, the Netherlands, Persia, Portugal, Roumania, Russia, Servia, Switzerland, Egypt, and the United States, is as follows:

ARTICLE 1. Each government shall immediately notify the other governments of the first appearance in its territory of authentic cases of plague or cholera.

Particulars are required, constant information provided, and preventive measures showing the opinion of the experts of every nation as to the extreme importance of protecting the world against bubonic plague.

Yet our Marine-Hospital Bureau was prevented from making the truth known, and even in its publications made its notice as obscure as possible for several years. The bureau understood the importance of publishing the truth; the bureau desired to tell the truth, but it was suppressed. I refer to this painful history not to criticise the . . . . . . . . . . . . . bureau, but to point out the fatal weakness of a subordinated bureau as compared with the dignity and power of a department.

OBLIGATIONS TO AMERICAN REPUBLICS.

The first general International Sanitary Convention of the American Republics, held at the Willard Hotel, Washington, December 2, 4, 1902, adopted resolutions of the delegates providing a provisional program and emphasising the sanitary convention adopted by the Second International Conference of the American States held in the City of Mexico, October 22, 1901, to January 22, 1902.

The convention of January 22, 1902, approved by the duly authorised delegates of the United States, Mexico, Bolivia, Colombia, Costa Rica, Chile, Dominocan Republic, Ecuador, Salvador, Guatemala, Haiti, Honduras, Nicaragua, Peru, and Uruguay, pledged the representative governments to coöperate with each other toward maintaining efficient and modern sanitary conditions, and provided:

That each and all of their respective health organisations shall be instructed to notify promptly the diplomatic or consular representatives of the republics represented in this conference of the existence or progress within their several respective territories of any of the following diseases: cholera, yellow fever, *bubonic plague,* and any other serious pestilential outbreak.

That it shall be made the duty of the sanitary authorities in each port prior to sailing of the vessel to note on the vessel's bill of health the transmissible diseases which may exist in such port at that time.

The Surgeon-General of the United States Public Health and Marine-Hospital Service was president of the convention at Washington of December 2, 1902. Mexico, not having been properly advised of the existence of bubonic plague at San Francisco, as agreed by the international convention of January 22, 1902, Mazatlan was infected, because of such failure of the officers of the United States to honorably comply with this convention.

The apology made for our conduct in this matter by Edward Liceago, president of the superior board of health of the Republic of Mexico (see report, 1903-4, on Public Health, p. 11), says:

*The authorities of San Francisco, Cal.,* fearing that the quarantine restrictions would perhaps impose on their commerce a closure of foreign ports, *had carefully concealed the existence of plague* and had given clean bills of health to ships leaving that port.

This infection of Mazatlan in December, 1902, took place nearly a year after the United States was bound by the sanitary convention of January 22, 1902, at Mexico City, to give Mexico notice.

What apology shall we offer other nations for such a violation of our international obligations to Mexico? What shall we say to Peru, Colombia, Chile, and the other American Republics for this gross breach of public faith?

Will they be content when we say this matter was in the care of a subordinate little bureau, which was thoughtlessly overruled by a Secretary not in sympathy with such a subject-matter? What shall we say to the state boards of health of Texas, Indiana, Colorado, and other state boards that demanded the report of the experts of the Marine-Hospital Bureau, and were denied the truth as to the bubonic plague in California?

Mr. President, a simple bureau will not do! It has been tried in the balance and found wanting.

The importance of the subject-matter, the dignity and honor of the United States, its international agreements, and the health and welfare of the world demand a department and a secretary of public health.

### TUBERCULOSIS.

Mr. President, Frederick L. Hoffman, statistician of the Prudential Life Insurance Company (Statistical Laws of Tuberculosis, *American Medical Journal,* 1904), estimates the commercial loss per annum to the United States from tuberculosis alone at $240,000,000.

Collier's editorial ("Expressed in Money," July 25, 1908) estimates the loss from tuberculosis alone at $330,000,000 per annum, and says:

> Is it any wonder, then, that the best physicians are heart and soul engaged in the study of its prevention?

Mr. Hoffman ("Physical and medical aspects of labor and industry," Annals of the American Academy of Political and Social Science, May, 1906) endeavors to establish the approximate measure of the social and economic value of life, and estimates that fifty active years of a working man's life represents a total of $15,000. If death should occur at the age of 25, the economic loss to society would be $13,695; at 35, $10,395; at 50, $4,405.

I doubt if any member of the Senate would regard this measure of economic value as excessive, yet this estimate would make our preventable death loss equal an annual charge of over $6,000,000,000. The annual loss from tuberculosis is a hundred and fifty thousand lives to the United States at the average age of 35 years, a terrific social and economic loss. Most of this loss could be avoided.

SAVING OF LIFE IN NEW YORK.

I submit a table of the department of health of the city of New York, showing the general death rate from 1886 to 1908, improving from 25.99 to 16.52 per thousand, nearly 10 to the thousand and an improvement of nearly 40 per cent. (Exhibit 2.)

The tuberculosis death rate has improved from 4.42 to the thousand to 2.29 to the thousand, a like improvement.

In Paris the death rate from tuberculosis is twice as great, but, Mr. President, death from tuberculosis in Greater New York alone in 1908 was 10,147 persons, and from all causes 72,072. (Exhibit 3.)

The vast improvement which has been made in the saving of life is clearly shown from the tables to which I call the attention of the Senate. (Exhibit 4.)

I submit, also, Table No. 3, showing a great improvement in the death rate of children under 1 year of age during the summer months, from 1891 to 1909, in which the death rate has been decreased one-half. (Exhibit 5.)

I submit Exhibit No. 6, the method of the department of health, in controlling tuberculosis.

I particularly desire to submit to the Senate for their physical inspection certain maps showing the number of cases of tuberculosis in certain down-town sections of New York City, in the Cherry and Market streets quarter and Cherry and Pearl streets neighborhood and the immense improvement obtained by a few years of effort. (Exhibits 7, 8, and 9.)

You will observe that in one house as many as 22 cases of tuberculosis is reported. Such a section of a great city may be properly described as a charnal house, where the poor are denied a fair opportunity of life by the grinding processes of unthinking commercial energy and power, and are dying by thousands when they might be saved to the great economic gain of the United States, to the great financial and commercial advantage of this Nation. I do not make an appeal on the basis of humanity and patriotism alone, but I put it upon the cold basis that ought to appeal to the commercial instinct of the Nation, even if it seems to have forgotten the value of human life and of human happiness.

PRESENT COST OF HEALTH AGENCIES OF UNITED STATES.

The United States made appropriations for the present fiscal year for sanitary and health purposes in the following months, as nearly as I can ascertain:

| | |
|---|---|
| Department of Commerce and Labor | $ 533.000.00 |
| Navy | 1,827,428.00 |
| War | 6,400,734.00 |
| Treasury | 2.512,733.00 |
| Interior | 1,748,350.00 |
| Agriculture | 1,275,820.00 |
| State | 3.405.79 |
| Bureau of Public Printer | 7,270.00 |
| District of Columbia | 663,680.00 |
| Total | $14,972,320.79 |

A total of nearly fifteen millions. This does not include the service in the Philippine Islands, Porto Rico, nor Cuba, nor 114 physicians, nor 28 nurses among the Indians, nor the one hundred and odd clerks in the medical division of the Pension Office, nor the medical attention to sick prisoners, nor for the collection of medical statistics by the Census Bureau.

There appear to be over 12,000 persons employed in this service, not including those engaged in Porto Rico, Cuba, Panama, the Philippines, nor in the Agricultural Department.

These agencies ought to be consolidated in one bureau. It meets the best opinion in the United States.

The people of the United States are ready to support a department of public health and will indorse this general policy of concentrating all of the health agencies of government. "A department of public health" has been indorsed by the National Grange (Des Moines, 1909); by the American Federation of Labor, with about 2,000,000 members; by the American Medical Association, with about 80,000 physicians and surgeons affiliated; by the National Child-Labor Committee; by the Conferences of Governors; and in one form or another by every political platform.

The Republican platform for 1908 says:

We commend the efforts made to secure greater efficiency in national public-health agencies and favor such legislation as will effect its purpose.

The Ohio Republican platform of this year declared in favor of—

The organisation of all existing national public health agencies into a single national public-health department.

In Connecticut and other States similar declarations have been made.

The Democratic platform in 1908 in like manner states:

We advocate the organization of all existing national public-health agencies into a national bureau of public health, with such power over sanitary conditions connected with factories, mines, tenements, child labor, and such other conditions, connected within jurisdiction of Federal Government—and which do not interfere with the power of the States controlling public-health agencies.

The Committee of One Hundred of the American Association for the Advancement of Science and the American Medical Association, with 80,000 members, advocate a plank in a national platform in sentiment as follows:

Believing a vigorous, healthy population to be our greatest national asset, and that the growth, power, and prosperity of the country depends primarily upon the physical welfare of its people and upon their protection from preventable pestilences of both foreign and domestic origin and from all other preventable causes of disease and death, including the sanitary supervision of factories, mines, tenements, child labor, and other places and conditions of public employment or occupation involving health and life, we advocate the organisation of all existing national public-health agencies into a national *department of public health*, with such powers and duties as will give the Federal Government control over public-health interests not conserved by and belonging to the States, respectively.

THE CONSERVATION OF LIFE, HEALTH, AND EFFICIENCY.

I believe in the conservation of our natural resources—of our coal fields, oil and gas fields, water powers, forests, and mines; the development of our natural resources in establishing good roads and improving our waterways.

The conservation of these great natural resources of our national wealth are of great importance, but the conservation of the life of our people is of far greater importance, and the conservation of the vitality and efficiency of our people is a problem of the first magnitude, demanding immediate intelligent attention.

I earnestly invite the Senate to consider Senate bill No. 6049 and the Report on National Vitality, by the Committee of One Hundred on National Health, which has been published as a Senate document and which gives in a compact form the essential principles relative to this matter, an abstract and summary of which I insert as Exhibit 1.

Under a department of public health these problems can be worked out with far greater efficiency. The coöperation of the authorities of the several States of the Union and of the municipalities of the several States, each one operated along the lines of constitutional propriety, can be established by a department of public health with much greater efficiency than through a subordinate bureau.

Indeed, under a subordinate bureau such coöperation is impracticable. The bureau has not sufficient dignity or power in an emergency. It has no national standing. It can not take the initiative, but must always stand subject to the orders of a Secretary too greatly influenced by mere apparent commercial and fiscal interest. A bureau of public health so controlled is pitiful, if not despicable, as an agency of an enlightened Nation.

Mr. President, I present this matter to the Senate with no pride of authorship, because I deserve no credit in that respect, and am perfectly willing to assist a bill drawn by any other Senator which shall better accomplish the purposes which I have at heart.

I realize that my colleagues are intensely preoccupied with the multitude of demands upon their time and attention.

But this is a question of vast national importance. In eight years we have increased our expenditures over the average of preceding years by the huge sum of one thousand millions for the army and navy, and are spending 70 per cent. of the national income to cover the obligations of past wars and the preparation for possible future war, or about seven hundred millions per annum. But for war on preventable disease now costing us infinite treasure in life, efficiency, and commercial power and prestige we spend nothing and do not even employ the agencies we have in an efficient manner.

In the name of the people and in the name of the American Medical Association, whose members are the faithful and self-sacrificing guardians of the health of our people, and in the name of the Committee of One Hundred, of the American Federation of Labor, of the National Grange, and of the various health boards of the 46 States of the Union and of the great body of learned men desiring improved sanitation and the application of the improved agencies of preventing disease, disability and death, I pray the Senate to establish a department of public health.

The principle of the bill meets the general approval of the public health societies and of the medical associations of the United States, and there should be no difficulty in perfecting this bill and in impressing upon the country the importance of organised effort to control the ravages of tuberculosis, typhoid and malarial fevers, bubonic plague, and other preventable diseases, which inflict such enormous injury upon the people of the United States, impose such vast, but needless, human misery and pain, with great financial loss and loss of prestige and power.

A commercial nation will not be unmindful of the commercial value of the saving of life and efficiency possible, which is worth $3,000,000,000 per annum. A humane nation will not fail to act when it is known that we could save the lives of 600,-000 of our people annually, prevent the sickness of 3,000,000 of people per annum, who now suffer from preventable disease, and greatly abate the volume of human pain, misery and death.

I trust, Mr. President, that the Senate may not fail to take action in regard to this matter at the present session.

